# Preoperative White Blood Cell Count and Risk of 30-Day Readmission after Cardiac Surgery

**DOI:** 10.1155/2013/781024

**Published:** 2013-07-18

**Authors:** Jeremiah R. Brown, R. Clive Landis, Kristine Chaisson, Cathy S. Ross, Lawrence J. Dacey, Richard A. Boss, Robert E. Helm, Susan R. Horton, Patricia Hofmaster, Cheryl Jones, Helen Desaulniers, Benjamin M. Westbrook, Dennis Duquette, Kelly LeBlond, Reed D. Quinn, Patrick C. Magnus, David J. Malenka, Anthony W. DiScipio

**Affiliations:** ^1^The Dartmouth Institute for Health Policy and Clinical Practice, Departments of Medicine Section of Cardiology and Community and Family Medicine and Dartmouth-Hitchcock Medical Center, Lebanon, NH 03756, USA; ^2^Edmund Cohen Laboratory for Vascular Research, The University of the West Indies, Bridgetown, Barbados; ^3^Department of Surgery, Concord Hospital, Concord, NH, USA; ^4^Department of Surgery, Dartmouth-Hitchcock Medical Center, Lebanon, NH 03756, USA; ^5^Department of Surgery, Portsmouth Regional Hospital, Portsmouth, NH, USA; ^6^Department of Surgery, Central Maine Medical Center, Lewiston, ME, USA; ^7^Department of Surgery, Eastern Maine Medical Center, Bangor, ME, USA; ^8^Department of Surgery, Maine Medical Center, Portland, ME, USA; ^9^Department of Surgery, Catholic Medical Center, Manchester, NH, USA

## Abstract

Approximately 1 in 5 patients undergoing cardiac surgery are readmitted within 30 days of discharge. Among the primary causes of readmission are infection and disease states susceptible to the inflammatory cascade, such as diabetes, chronic obstructive pulmonary disease, and gastrointestinal complications. Currently, it is not known if a patient's baseline inflammatory state measured by crude white blood cell (WBC) counts could predict 30-day readmission. We collected data from 2,176 consecutive patients who underwent cardiac surgery at seven hospitals. Patient readmission data was abstracted from each hospital. The independent association with preoperative WBC count was determined using logistic regression. There were 259 patients readmitted within 30 days, with a median time of readmission of 9 days (IQR 4–16). Patients with elevated WBC count at baseline (10,000–12,000 and >12,000 mm^3^) had higher 30-day readmission than those with lower levels of WBC count prior to surgery (15% and 18% compared to 10%–12%, *P* = 0.037). Adjusted odds ratios were 1.42 (0.86, 2.34) for WBC counts 10,000–12,000 and 1.81 (1.03, 3.17) for WBC count > 12,000. We conclude that WBC count measured prior to cardiac surgery as a measure of the patient's inflammatory state could aid clinicians and continuity of care management teams in identifying patients at heightened risk of 30-day readmission after discharge from cardiac surgery.

## 1. Introduction

Approximately one in every five hospitalized patients is readmitted within 30 days [1]. Currently, two-thirds of US hospitals have reimbursement penalties for higher than expected 30-day readmission rates from the Center for Medicaid and Medicare Services [2, 3]. It is expected that similar penalties will be extended to other procedures and diagnoses including cardiac surgery. In preparation for the expansion of the penalty system in the USA and to improve prediction of patients at high risk of postdischarge complications leading to readmissions or premature death, risk factors must be identified early in the hospital course to align the best possible quality and continuity of care. 

Currently, a validated risk model for predicting readmissions after cardiac surgery is not available and few risk factors for readmission are known. Recent evidence from California reported an association between infection and higher rates of 30-day readmission after cardiac surgery [4]. However, identification of infection after discharge without routine monitoring of a postcardiac surgical patient is problematic. What is needed is for clinical care teams to identify patients at high risk of infection before cardiac surgery to determine readiness and safety for the patient to undergo surgery. A common marker of inflammation is white blood cell (WBC) count, routinely measured prior to cardiac surgery. WBC count provides a broad measure of inflammation status, whether as a result of infection or proinflammatory disease states such as diabetes, COPD, or hemodialysis [5–8]. Elevated WBC count is reported as a component of the systemic inflammatory response syndrome (SIRS) to sepsis and is endorsed as a marker for reporting the systemic inflammatory response to cardiopulmonary bypass [9, 10]. In addition, current evidence has shown that preoperative WBC count is predictive of in-hospital mortality and stroke [11] and major bleeding [12] after coronary artery bypass graft surgery and associated with complications in other endovascular and thoracic procedures [13, 14], suggesting that preoperative WBC count may aid clinical care teams in risk-stratifying patients prior to surgery. However, it is not known if a patient's baseline inflammatory state measured by crude WBC count could predict 30-day readmission. Therefore, we sought to evaluate whether preoperative WBC count was associated with 30-day readmissions after cardiac surgery. 

## 2. Methods

Patients undergoing coronary artery bypass graft (CABG) surgery and/or valve surgery within the Northern New England Cardiovascular Disease Study Group (NNE) between July 2008 and December 2010 were enrolled in the cohort. A total of 2,209 consecutive patients were included along with 268 readmissions to the hospital performing the index cardiac surgery. Twelve patients were excluded due to missing white blood cell counts and twenty-one for incomplete data, leaving a total of 2,176 patients and 259 readmissions occurring within 30 days of discharge from the index cardiac surgery admission. All institutional review boards for each center reviewed and approved the data collection for the NNE registry and supplementary data collection for readmissions.

The NNE is a voluntary regional consortium of physicians, allied health professionals, research scientists, and hospital administrators from institutions in Maine, New Hampshire, and Vermont that support coronary revascularization and open-heart surgery. The goal of the consortium is to foster continuous improvement in the quality, safety, and effectiveness of care for patients with cardiovascular disease through the analysis of process and outcomes data with timely feedback to the health care professionals providing these services. All the hospitals providing open-heart surgery in this region contribute data on consecutive cases with validation of procedure numbers and mortality performed every two years. The registry collects data on patient characteristics, procedural indication, priority, and process, and in-hospital outcomes (see http://www.nnecdsg.org/ for the data forms and publically available data). 

WBC count was defined as the last preoperative measurement of WBC taken prior to procedure, was collected by data abstractor at each center. Categories of WBC counts were divided into predefined categories (<6.0, 6.0–7.9, 8.0–9.9, 10.0–12.0, and >12.0 thousands per cubic millimeter, mm^3^). 

Baseline, operative, and postoperative outcomes were compared using chi-square tests and continuous data using Student's *t*-test or Wilcoxon rank sum tests where appropriate. We conducted both univariate and backwards stepwise logistic regression removing risk factors that did not reach an alpha <0.1 among only risk factors with an alpha <0.1 from univariate comparisons. All risk factors meeting an alpha <0.1 were included in the final model multivariate logistic regression model. Categories of white blood cell counts were then added to the multivariate clinical risk prediction model. We conducted a Hosmer-Lemeshow goodness of fit test and calculated the area under the receiver operating characteristic (ROC) curve for the final multivariate model with categories of white blood cell count and reported the ROC and 95% confidence intervals for each model. All analyses were performed using Stata 11.2 (College Station, TX).

## 3. Results

Among the 2,176 patients, 259 patients were readmitted within 30 days (11.9%). The median time of readmission was 9 (IQR 4–16) days. Patient demographics were similar between patients with a 30 day readmission and those without a readmission. Patients readmitted within 30 days were more likely to have chronic obstructive pulmonary disease, history of dialysis, single vessel coronary disease, and white blood cell counts greater than ten thousand prior to surgery (Table 1). Procedural factors associated with 30-day readmission included valve or combined CABG/valve procedure, on-pump surgery, nadir hematocrit <20 on bypass, three or more packed red blood cell transfusions, use of inotropes, and the development mediastinitis, AKI, or atrial fibrillation (Table 2).

**Figure 1:**
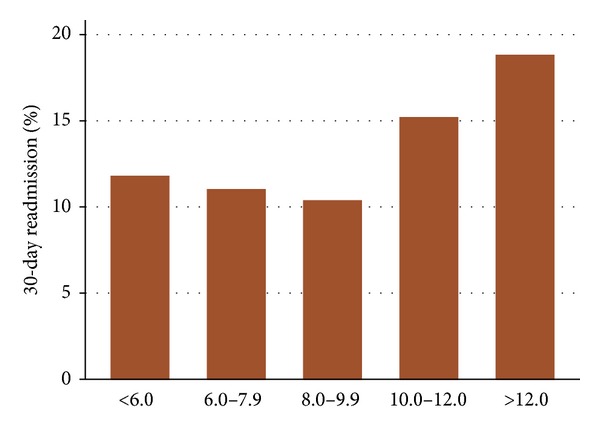
Preoperative White Blood Cell Counts and Risk of 30-day Readmission. The graph plots the risk of all-cause 30-day readmission by five pre-defined categories of preoperative white blood cell counts (in thousands per cubic millimeter, mm^3^).

Patients with elevated WBC counts at baseline (10,000–12,000 and >12,000 mm^3^) had higher 30-day readmission than those with lower WBC counts prior to surgery (15% and 18% compared to 10%–12%, *P* = 0.037, Figure 1). After backwards stepwise regression, WBC count and other risk factors remained significantly associated with 30-day readmission including number of diseased vessels, on-pump surgery, nadir hematocrit <20 on bypass, receiving three or more packed red blood cells, developing mediastinitis, and acute kidney injury (Table 3). Type of surgery (valve, isolated coronary artery bypass graft, or combined valve/graft) and duration of bypass were not significantly associated with readmissions in the multivariate model. Adjusting odds ratios for preoperative WBC counts were 1.42 (0.86, 2.34) for counts 10,000–12,000 (mm^3^) and 1.81 (1.03, 3.17) for counts >12,000 (mm^3^) (Table 2). The calculated c-statistic was 0.66 with a Hosmer-Lemeshow goodness of fit chi-square of 10.94 and *P* value of 0.2. Patient and procedural characteristics stratified by white blood cell categories are summarized in Table 4.

## 4. Discussion

We explored the predictive ability of WBC counts prior to cardiac surgery on 30-day readmission. With and without adjustment of other risk factors for readmission, patients with preoperative WBC counts >12,000 (mm^3^) were significantly more likely to be readmitted to the hospital within 30 days from discharge. We are the first to demonstrate that a marker of inflammation prior to the start of surgery demonstrates increased risk of 30-day readmission and should be incorporated into risk models to predict readmission prior to discharge from cardiac surgery.

WBC count has enjoyed a resurgence in recent years as a valid marker of inflammation and as a strong independent predictor of future coronary heart disease and stroke [15, 16]. After an acute event, patient outcomes remain influenced by WBC count at the time of hospital admission. In several studies, peak WBC count or elevated monocyte count has been linked to death or major adverse cardiac events (MACEs) outcomes, including readmission [17–19]. Other strong evidence has linked high WBC count at admission with adverse outcomes (mortality and bleeding) in patients undergoing coronary revascularization with cardiopulmonary bypass [11, 12]. However, in the case of cardiopulmonary bypass it is unclear whether high WBC count contributes to preexisting risk or to development of the systemic inflammatory response postoperatively or both.

The systemic inflammatory response is a complication in cardiopulmonary bypass patients that is caused by a combination of surgical stress and contact activation of blood component in the extracorporeal circuit [20, 21]. It is poorly defined [22] and the only formal definition is the Systemic Inflammatory Response Syndrome (SIRS), borrowed from the sepsis field [9]. According to the definition, SIRS exists when any two out of four criteria relating to abnormal temperature, heart rate, respiratory rate, or white cell counts exist. The upper threshold for abnormal white cell count according to the definition is 12,000 [9]. An evidence-based review of the inflammatory response indicated that all four SIRS criteria were rarely monitored in the setting of cardiopulmonary bypass [23] as they were felt to be too nonspecific [22] and if taken literally would apply to approximately 40% of all patients [24–26]. A more recent update on minimal reporting criteria by the Outcomes Consensus Panel singled out WBC count as the only criterion measured on its own as being relevant to the inflammatory status [10]. This recommendation was supported by other fields in which WBC count is recognized as a valid marker of inflammation [5, 6, 8, 27].

An alternative theory for the development of the systemic inflammatory response is that this is determined less by the extracorporeal circuit itself but rather by preexisting activation of white cells and endothelium [28] or by preoperative transfusion. Consistent with this theory is that high WBC count prior to coronary surgery utilizing cardiopulmonary bypass is linked with adverse outcomes including mortality and bleeding [11, 12]. Our present findings that high WBC count before-surgery is linked to an increased risk of 30-day readmission after discharge add further weight to this idea. We therefore conclude that WBC count measured prior to cardiac surgery may serve as a measure of the patient's inflammatory status and could aid in identifying and managing patients at heightened risk of readmission after discharge from cardiac surgery. This becomes especially relevant in an era when higher than expected readmission rates may attract financial penalties to hospitals.

## Figures and Tables

**Table 1:** Characteristics of patients with or without 30-day readmission.

Characteristic	30-day readmission		
	No	Yes	*P* value
Number of patients (2,176)	1,917	259	
Demographics			
Age	66.2 ± 11.2	66.4 ± 11.5	0.740
Female	28.5	32.8	0.154
BMI	29.6 ± 5.9	29.8 ± 6.5	0.585
Comorbidities			
Type 2 diabetes mellitus	31.5	37.1	0.070
Vascular disease	27.3	30.9	0.224
COPD	15.6	20.5	0.043
History of dialysis	2.4	5.0	0.015
Smoking	21.4	22.4	0.726
Cardiac history			
Recent MI	17.3	13.1	0.091
CHF	19.9	25.1	0.051
Prior CABG	3.9	2.7	0.337
Prior valve	1.5	1.9	0.611
Prior PCI	18.2	17.4	0.760
NYHA Class IV	15.6	15.4	0.949
Cardiac anatomy and function			
Left main disease ≥50%	28.8	20.9	0.007
Single-vessel disease	33.5	42.6	0.002
Two-vessel disease	28.5	30.2	
Three-vessel disease	38.1	27.3	
Ejection fraction			
<40%	11.3	12.3	0.894
40%–59%	12.6	11.1	
50%–59%	24.3	24.2	
≥60%	51.7	52.4	
White blood cell count (in 1,000's, mm^3^)			
<6.0	19.9	19.7	0.037
6.0–7.9	39.2	35.9	
8.0–9.9	25.3	21.6	
10.0–12.0	9.9	13.1	
>12.0	5.6	9.7	

COPD: chronic obstructive pulmonary disease; MI: myocardial infarction; CHF: congestive heart failure; CABG: coronary artery bypass graft surgery; PCI: percutaneous coronary intervention; WBC: white blood cell; eGFR: estimated glomerular filtration rate.

**Table 2:** Procedural characteristics and outcomes of patients with or without 30-day readmission.

Characteristic	30-day readmission		
	No	Yes	*P* value
Procedural characteristics			
Priority			
Emergent	5.3	6.6	0.534
Urgent	50.9	47.9	
Elective	43.9	45.6	
Procedure			
CABG	60.7	51.0	0.007
Valve	22.4	30.1	
CABG/valve	17.0	18.9	
On-pump surgery	90.3	95.8	0.004
Nadir hematocrit <20 on bypass	15.0	20.5	0.029
Cardiopulmonary bypass time (min)	119.7 ± 53.7	122.9 ± 56.5	0.393
Time to initial extubation (min)	17.3 ± 65.2	17.6 ± 35.1	0.951
Intraoperative myocardial infarction	2.4	2.7	0.725
Return to bypass	4.2	4.6	0.730
Management			
RBC transfusions			
None	67.6	58.7	0.001
One	8.9	10.0	
Two	10.3	9.3	
Three or more	13.2	22.0	
Use of 1 or more inotropes			
Arrive to ICU	43.9	44.0	0.965
After 4 hours	37.2	44.0	0.034
After 48 hours	11.3	15.1	0.080
Adverse outcomes			
Low-cardiac output failure	8.0	10.0	0.272
Stroke	1.1	1.5	0.525
Mediastinitis	0.4	2.7	<0.001
Acute kidney injury	30.7	49.4	<0.001
Reintubation	3.7	3.9	0.866
Return to operating room for bleeding	3.2	4.3	0.395
New atrial fibrillation	32.6	39.8	0.021
Leg wound infection	0.7	1.2	0.463
Pneumonia	1.7	0.8	0.275

CABG: coronary artery bypass graft surgery; RBC: packed red blood cell transfusion; ICU: intensive care unit.

**Table 3:** Univariate and multivariate regression anaylsis for 30-day readmission.

	Odds ratios (95% CI) for 30-day readmission	
	Univariate	Multivariate
White blood cell count (in 1,000's, mm^3^)		
<6.0	Reference	Reference
6.0–7.9	0.93 (0.64, 1.33)	0.96 (0.65, 1.41)
8.0–9.9	0.86 (0.58, 1.29)	0.91 (0.59, 1.39)
10.0–12.0	1.34 (0.84, 2.14)	1.42 (0.86, 2.34)
>12.0	1.73 (1.03, 2.93)	1.81 (1.03, 3.17)
Other risk factors		
Single-vessel disease	1.77 (1.28, 2.46)	1.73 (1.24, 2.43)
Two-vessel disease	1.48 (1.04, 2.10)	1.43 (1.00, 2.06)
On-pump surgery	2.43 (1.31, 4.54)	1.85 (0.98, 3.52)
Nadir hematocrit on bypass <20	1.55 (1.10, 2.17)	1.39 (0.96, 2.00)
Three or more packed red blood cells	1.86 (1.34, 2.56)	1.52 (1.07, 2.18)
Mediastinitis	7.58 (2.64, 21.79)	5.81 (1.87, 18.08)
Acute kidney injury	2.21 (1.70, 2.87)	2.03 (1.53, 2.68)
Model parameters		
Hosmer-Lemeshow *χ* ^2^, *P* value		*χ* ^2^ = 10.94, *P* value = 0.2
ROC		0.66

WBC: white blood cell; SD: standard deviation of the log-transform of WBC count; ROC: area under the receiver operating characteristic curve.

**Table 4:** Characteristics of patients and white blood cell count categories.

Characteristic	White blood cell count (in 1,000s)					
	<6.0	6.0–7.9	8.0–9.9	10.0–12.0	>12.0	*P* value
Number of patients (2,176)	433	845	541	224	133	
Demographics						
Age	67.7 ± 11.3	67.1 ± 10.6	65.5 ± 11.4	63.6 ± 11.7	63.5 ± 12.1	<0.001
Female	30.3	29.5	26.6	31.7	27.8	0.594
BMI	28.5 ± 5.3	29.6 ± 6.0	30.3 ± 6.2	30.3 ± 6.1	29.4 ± 5.5	<0.001
Comorbidities						
Type 2 diabetes mellitus	26.3	31.6	36.8	32.6	34.6	0.014
Vascular disease	25.6	27.9	27.9	29.9	28.6	0.817
COPD	12.2	14.7	19.4	20.1	18.1	0.009
History of dialysis	2.1	2.4	2.6	2.7	7.5	0.013
Smoking	9.7	17.9	28.8	35.7	30.1	<0.001
Cardiac history						
Recent MI	7.6	15.5	17.6	28.1	33.1	<0.001
CHF	18.7	18.7	21.4	22.3	30.8	0.017
Prior CABG	5.1	3.9	3.1	1.8	4.5	0.256
Prior valve	2.5	1.3	0.9	1.3	3.0	0.173
Prior PCI	15.9	18.6	19.2	17.0	18.8	0.699
NYHA Class IV	12.0	12.2	16.6	24.6	29.3	<0.001
Cardiac anatomy and function						
Left main disease ≥50%	22.9	27.9	27.9	31.3	37.6	0.012
Single-vessel disease	41.6	34.7	33.0	28.1	26.8	0.013
Two-vessel disease	27.5	28.2	28.8	32.9	28.5	
Three-vessel disease	30.9	37.2	38.2	39.1	44.7	
Ejection fraction						
<40%	9.4	9.3	12.0	15.0	22.8	<0.001
40%–59%	11.1	10.9	14.1	18.2	9.5	
50%–59%	25.9	24.2	24.1	21.5	26.0	
≥60%	53.5	55.5	49.8	45.3	41.7	
Procedural characteristics						
Priority						
Emergent	1.6	2.8	6.1	10.3	23.3	<0.001
Urgent	46.0	47.9	51.2	61.2	60.9	
Elective	52.4	49.2	42.7	28.6	15.8	
Procedure						
CABG	50.6	59.1	62.5	67.4	66.2	<0.001
Valve	31.4	23.7	21.3	15.2	16.5	
CABG/valve	18.0	17.3	16.3	17.4	17.3	
On-pump surgery	92.4	92.0	88.7	89.7	90.2	0.213
Nadir hematocrit <20 on bypass	11.8	15.0	13.7	12.1	15.8	0.465
Cardiopulmonary bypass time (min)	121.5 ± 55.2	117.1 ± 49.2	122.1 ± 55.7	121.4 ± 62.2	124.7 ± 57.9	0.656
Time to initial extubation (min)	14.4 ± 28.6	15.6 ± 32.7	16.6 ± 51.4	20.8 ± 51.8	35.9 ± 199.8	<0.001
Intraoperative myocardial infarction	1.2	2.5	2.4	3.1	4.5	0.203
Return to bypass	5.1	4.7	3.0	3.1	5.3	0.343
Management						
RBC transfusion	36.3	32.9	30.7	31.7	43.6	0.041
Use of 2 or more inotropes within 48 hours	2.1	3.6	2.5	4.5	4.6	0.278
Adverse outcomes						
Low-cardiac output failure	7.4	8.8	6.7	8.5	14.3	0.063
Stroke	1.2	0.8	1.5	1.8	0.8	0.687
Mediastinitis	0.2	0.7	0.6	0.9	1.5	0.552
Acute kidney injury	29.3	33.6	31.4	37.5	38.4	0.128
Reintubation	3.0	2.8	4.8	4.5	5.3	0.239
Return to operating room for bleeding	6.0	3.1	1.9	2.2	4.5	0.005
New atrial fibrillation	32.1	34.8	31.8	32.6	36.8	0.650
Leg wound infection	0.7	0.8	0.7	1.3	0.0	0.731
Pneumonia	1.6	1.4	1.1	3.1	1.5	0.354

COPD: chronic obstructive pulmonary disease; MI: myocardial infarction; CHF: congestive heart failure; CABG: coronary artery bypass graft surgery; PCI: percutaneous coronary intervention; WBC: white blood cell; eGFR: estimated glomerular filtration rate; CABG: coronary artery bypass graft surgery; RBC: red blood cell; ICU: intensive care unit.
